# Carbonic Anhydrase as a Sustainable Sealing Agent for Concrete Exposed to Chloride Attack

**DOI:** 10.1021/acssuschemeng.5c01165

**Published:** 2025-06-30

**Authors:** Pardis Pourhaji, Nima Rahbar

**Affiliations:** Department of Civil, Environmental and Architectural Engineering, 8718 Worcester Polytechnic Institute, 100 Institute Road, Worcester, Massachusetts 01609, United States

**Keywords:** enzymatic self-sealing concrete, service life, carbonic anhydrase, chloride penetration, sustainability

## Abstract

Concrete, the most used engineering material in the world, is responsible for 8% of the entire CO_2_ emission; 80% of the failures in reinforced concrete structures are related to the corrosion of embedded reinforcement. Concrete resistivity against chloride penetration is a pivotal parameter in determining the durability of reinforced concrete structures. Self-sealing concrete has recently emerged as a powerful method for enhancing this resistance. This paper investigates the effect of carbonic anhydrase (CA) as a sealing agent on the chloride penetration of concrete by the rapid chloride penetration test (RCPT). Concrete disk specimens without CA and with two different dosages of CA were cast and cured for 14 and 28 days. Chloride penetration depths and migration coefficients were evaluated for uncracked specimens at 14 and 28 days. The lower dosage of CA was used to prepare cracked and sealed specimens. The chloride content was quantified, and service life was predicted for specimens at 28 days. In uncracked concrete, a lower dosage of CA can considerably increase concrete resistivity to chloride penetration up to 46% compared to the control specimens. Although the presence of a crack increased chloride penetration through the crack, sealing the crack showed penetration depth at the crack zone similar to the uncracked concrete. Hence, using CA enzyme as a sealing agent extends the service life of uncracked concrete and recovers the cracked concrete’s service life after sealing. Thus, the addition of enzymes will have significant implications for infrastructure sustainability and reduction of CO_2_ emissions related to the second industrial emitter.

## Graphical abstract

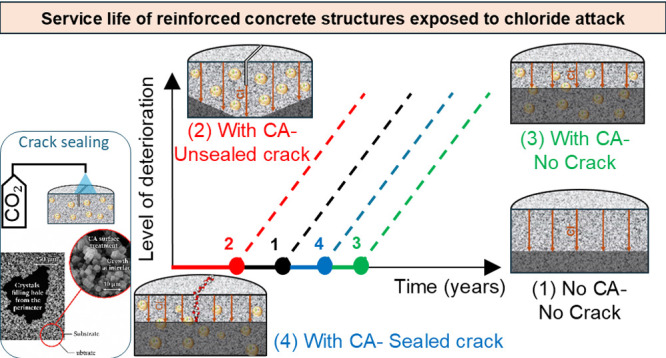

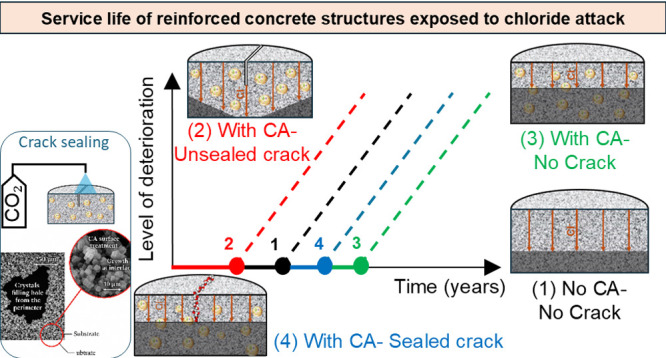

## 1 Introduction

Corrosion of reinforcement is a major threat to the service life of concrete structures, causing civil engineers significant concern due to its potential to reduce durability and structural integrity. Concrete, which is the most used material, is inherently weak in tension. Many studies have been carried out to improve the tensile properties, such as adding polymer fibers^1^ to cementitious materials. Most importantly, the use of concrete comes at an environmental cost. The environmental impact of maintaining and repairing concrete infrastructure is substantial from a sustainability point of view. Almost 8% of human-made global carbon emissions and 3% of global energy demand come from the production of concrete materials.^2–4^

One of the main factors in the corrosion of reinforcing bars in concrete structures and the corresponding concrete deterioration is the transfer of harmful substances, such as chloride, via water or gas. Diffusion and permeation are the main mechanisms to transport these substances in concrete,^5^ which is predominantly controlled by the pore structure. Connectivity, size, and distribution of the pores in the cementitious matrix play essential roles in the permeability of concrete.^6^ Most corrosion inhibitors, such as calcium nitrite, can increase chloride ion transport, slightly reduce the long-term compressive strength, and shorten the setting time of concrete at higher dosages.^7^ Additionally, they pose significant toxicity concerns, making them less environmentally friendly than other alternatives.^8^

Although water permeability is an indicator of the transport properties of concrete, the diffusion mechanisms play the primary role when salt or CO_2_ are present. Accumulated chloride above a critical diffused content expedites the corrosion of embedded reinforcement in concrete.^9^ Moreover, the presence of cracks aggravates the situation by making a shorter path of transporting harmful agents into the concrete.^6^

Based on the thermodynamic model of Tuutti,^10^ the service life of concrete is divided into two main phases: initiation and propagation. Aggressive agents, such as chloride ions, penetrate from the concrete cover to the rebar surface at the initiation phase. When chloride accumulation at the rebar level reaches a certain level, the rebar surface is depassivated and corrosion propagates. Concrete cover characteristics and environmental conditions are essential in the corrosion initiation phase, while the steel–concrete interface is the main factor in the propagation phase. Based on this model, studies are carried out to model the service life of different types of concrete,^11^ and standards are developed to design more durable concrete structures.^12,13^

Concrete is a porous composite containing both isolated and connected pores. The presence of a crack leads to a linkage of pores and an increase in concrete permeability. Hence, the deterioration of a cracked reinforced concrete element speeds up when exposed to an aggressive environment.^14^ Weiss et al.^14^ investigated the impact of crack width and length on chloride ingress in concrete with w/c ratios of 0.30, 0.42, and 0.50 and aggregate sizes of 4, 8, and 16 mm. It was found that as crack width and length increase, the crack behaves more like a free surface, thereby diminishing the influence of the mixture parameters. These findings highlight the complexity of chloride ingress in cracked systems and the importance of considering the crack geometry. Savija et al.^15^ discussed that chloride ingress is strongly influenced by surface cracking, with wider cracks promoting deeper and more extensive penetration. In a comparative study of OPC and slag concrete, wedge-splitting specimens with controlled crack widths (55–424 μm) were exposed to weekly salt (3.3% NaCl) wet–dry cycles, revealing significantly higher chloride penetration in the OPC with a wider crack. As a result, significant resources are needed to have more sustainable concrete structures.^16,17^ One pioneering approach to extend the lifespan of concrete structures with more sustainability is using self-sealing concrete.^18,19^

The self-sealing ability of concrete can be enhanced by the addition of external sealing agents to seal the crack autonomously.^20^ There are different types of proposed self-sealing mechanisms and agents.^21–23^ Encapsulation of sealing agents is one of the well-known methods widely used recently for sealing wide crack widths;^24^ however, the technique can only work once in the structure’s lifespan. Additionally, setting up real-scale demonstrations is complex, and the industry is cost-sensitive. Lauch et al.^25^ investigated the impact of various self-sealing agents, including crystalline admixtures, expansive agents, and superabsorbent polymers, on the water permeability of cracked concrete. A crack width of 0.25–0.3 mm was introduced into the concrete specimens. Specimens containing crystalline admixture were submerged in water for three months, while specimens with expansive agents and superabsorbent polymers underwent wet–dry cycles for 3 months to facilitate crack sealing. The findings demonstrated partial sealing of the cracks at the surface and a reduction in the water permeability. Moreover, it was observed that the addition of a crystalline admixture and superabsorbent polymer negatively affected the slump flow of the fresh concrete. Effects of biochar as a green additive were studied on the self-sealing cementitious matrices. A 60 μm crack width was introduced on the matrix, and then specimens were submerged partially under water for 2 weeks to seal the crack. The results showed that specimens containing biochar had higher Ca­(OH)_2_ and CaCO_3_ values than the control specimens. In addition, complete crack sealing at the surface occurred after 28 days. However, biochar reduced the compressive strength and electrical resistivity of the matrix and increased the total porosity compared with the specimens without biochar.^26^ The enzymatic method is the only technique that can rapidly seal cracks with widths in the range of 0.3–0.5 mm.

Aspiotis et al.^27^ studied the durability of self-sealing in ordinary Portland cement concrete containing chemical additives through a non-steady-state chloride migration test before and after crack sealing. Five different mix designs were used, incorporating control without additive (Mix 1), with commercial crystalline waterproofing cementitious product and an expansive additive (Mix 2), containing expansive additive (Mix 3), containing two different dicarboxylic acids, designated as unsaturated dicarboxylic acid and substituted dicarboxylic acid, expansive additive, and sodium carbonate (Mix 4), and mixed 4 with silica fume (Mix 5). Two groups of specimens were prepared: one as a reference (uncracked) and the other with artificially induced cracks running across both circular surfaces, with the halves reassembled and held together using a clamp. The crack widths ranged from 30 to 400 μm. In the applied sealing procedure, the specimens remained in water for 60 days at 20 ± 2 °C and efficient monitoring of the crack sealing evolution was ensured by assessing three positions marked on the surface of each specimen. The results indicated that a significant number of cracks were completely sealed in all of the specimens. Non-steady-state chloride migration tests were applied to both groups of samples in each concrete mix based on NT BUILD 492. The migration of chlorides was faster in the self-sealing specimens than in the uncracked concrete in each similar mix design. Xue^28^ studied the effect of cracking and autogenous self-sealing on the performance of fiber-reinforced MgO-cement composites exposed to seawater and NaCl solutions. Two concrete mixes were prepared, one with and one without commercially available light-burnt MgO and crystalline admixtures, both incorporating fibers to control the crack width. After 7 days of curing, a splitting test was applied to the cylindrical specimens to create a longitudinal crack with a width of 60 μm after unloading. The cracked and uncracked specimens were then subjected to two exposure conditions: continuous immersion and wet/dry cycling, simulating the marine tidal zone. They found that the MgO-based mix exhibited enhanced crack sealing, particularly under wet/dry cycles. After three months, chloride profiling revealed that the uncracked specimens containing MgO and crystalline admixtures had a slightly lower chloride content at each level. However, even fully sealed cracked specimens showed higher chloride levels than uncracked specimens under the same exposure conditions.

Carbonic anhydrase (CA) is a biological enzyme that catalyzes the chemical reaction between water, calcium ions (Ca^2+^), and CO_2_ to produce calcium carbonate (CaCO_3_) at a highly rapid rate without being consumed in the process.^29^ As CA is a catalyst and will not be consumed during the procedure,^30^ a small amount of CA can be used in the cementitious composite. As soon as a crack appears, it catalyzes the mentioned reaction when there is access to CO_2_, and CaCO_3_ precipitates at the crack zone and fills up the flaw.^31^ CA is the fastest known enzyme for this reaction, and the crystal growth rate using this method is orders of magnitude faster and more efficient than any other method, such as bacterial ones. At the record, this method can seal a 1 mm crack within 24 h.^19^ In this regard, there is a possibility of extending the service life of the infrastructure, while minimizing carbon emissions and energy consumption.

Here, we systematically investigate the effect of CA incorporation in mitigating chloride ingress in concrete using the Rapid Chloride Penetration Test (RCPT) on uncracked conventional concrete disk specimens without and with two different dosages of CA at 14 and 28 days of age. Then, we chose a more efficient dosage of CA to prepare cracked concrete disks. RCPT was then performed on cracked concrete containing CA before and after sealing for 28 days. Finally, the mechanisms of chloride diffusion are thoroughly examined and are discussed. The service life of uncracked and sealed cracked concrete specimens at 28 days is computed and provided, and a sustainability assessment based on quantitative environmental impact metrics is conducted.

## 2 Materials and Methods

To ensure consistency in our experiments, we maintained controlled environmental conditions throughout the testing process. The temperature and relative humidity (RH) in the laboratory were kept constant at 23 ± 2 °C and approximately 55 ± 5% RH, respectively, from specimen preparation to the conclusion of the tests. These conditions were carefully monitored, since they can influence the results and potential applicability of the sealing method in various real-world situations.

### 2.1 Material Properties

Concrete specimens with and without carbonic anhydrase were used for all of the analyses in this study. The cement composition was analyzed by X-ray fluorescence (XRF), and the results are shown in Table 1. Aggregate proportions were chosen based on a well-distributed grading. The fine aggregate includes two grading sizes of sand: 0 to 2 mm and 2 to 4 mm. Crushed aggregate was used with a grading between 4 and 12.5 mm for the coarse aggregates.

**Table 1:** Elemental Composition of Cement Powders by X-ray Fluorescence (XRF)

XRF	wt %	XRF	wt %	XRF	wt %
Si	42.24	Ca	36.94	Fe	5.3
Mg	5.18	Al	2.93	Pd	1.99
Zr	1.99	Ti	1.69	Te	0.73
Mo	0.29	Mn	0.28	Co	0.17
Cu	0.16	Ni	0.10	total	99.99

#### 2.1.1 Carbonic Anhydrase

From pure stoichiometry, the theoretical maximum CO_2_ consumption is 0.44 g of CO_2_ per gram of CaCO_3_ produced. In this paper, carbonic anhydrase (CA) is used as a sealing agent. The CA enzyme was supplied as a lyophilized powder from Millipore-Sigma (catalog number C2624, CAS Number: 9001–03–0 ^32^). CA is a harmless material for human health, decomposes without any risk, does not produce any harmful byproducts, and has the capacity for catalyzing crystal growth and plugging macroscale pores and cracks upon exposure to CO_2_. It can produce 220 CaCO_3_ g/L per day, where for every 1 g of CaCl_2_, 0.1 g of CO_2_ is consumed for every 1 g of CaCO_3_ produced based on the experimental uptake efficiency under the specific enzyme-catalyzed conditions. This estimation is derived from the observed pH change during the reaction, based on the assumption that two hydrogen ions are produced per molecule of CO_2_ consumed and the lower conversion yield (around 10%) is attributed to factors such as limited CO_2_ dissolution, buffering effects of the tris solution, and the short reaction duration.^19^ To estimate the required enzyme activity for the conversion of CO_2_ in the production of 220 g/L of CaCO_3_ per day, we first calculate the daily CO_2_ consumption. Given that the molar mass of CO_2_ is 44 mg/mmol, 22 g of CO_2_ corresponds to 500 mmol per day. Since one W-A unit catalyzes the conversion of 0.001 mmol of CO_2_ per minute under standard conditions, it processes 1.44 mmol of CO_2_ per day. To meet the CO_2_ consumption of 500 mmol per day, the number of W-A units required is calculated as 500 mmol/day divided by 1.44 mmol/day per unit, which equals approximately 347.2 W-A units. Considering that the enzyme activity used in our study is 3500 W-A units per mg of CA, the mass of CA needed to achieve this enzyme activity is 0.099 mg. Carbonic anhydrase is a zinc-containing metalloenzyme and contains pockets of amino acids His94, His96, and His119 holding the zinc ion (Figure 1).

**Figure 1:**
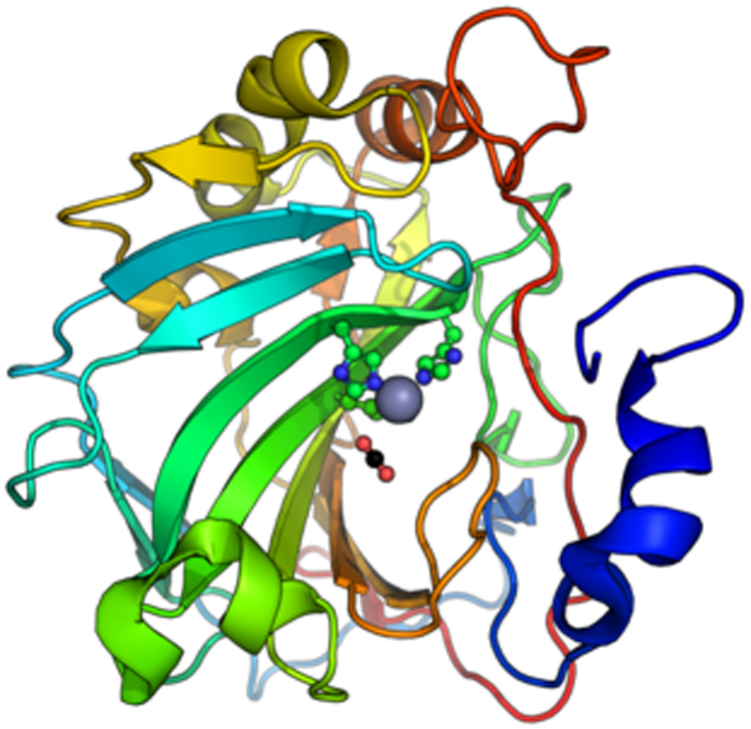
Protein structure of carbonic anhydrase contains a pocket of amino acids His94, His96, and His119 holding the zinc ion.^33^

It catalyzes a reaction converting CO_2_ and water into carbonic acid HCO_3_^–^ with gaining an OH^–^ which was bonded to the zinc when a CO_2_ enters the active site of the enzyme. Water dissociates to replenish the OH^–^, the OH^–^ ion binds to the zinc and the H^+^ ion is released (eqs 1 and 2). These reactions repeat.

1 $${\mathrm{CO}}_{2}+H_{2}O\overset{\mathrm{CA}}{\to }{\mathrm{HCO}}_{3}^{-}+H^{+}$$

2 $${\mathrm{Ca}}^{2+}+{\mathrm{HCO}}_{3}^{-}\to ↓{\mathrm{CaCO}}_{3}^{+}+H_{2}O$$

The hydration state of concrete significantly influences the efficiency of carbonic anhydrase-mediated reactions. When concrete is adequately hydrated, the pore water facilitates the dissolution of CO_2_ and enhances the reaction, leading to efficient crack sealing. However, in dry concrete, limited moisture availability can reduce the level of CO_2_ dissolution, thereby slowing or inhibiting carbonation and sealing processes. The depth and extent of carbonation also strongly depend on relative humidity (RH). The optimal ambient RH provides sufficient moisture for both the CO_2_ dissolution and precipitation of sealing products, ensuring effective crack sealing.

The stability of carbonic anhydrase (CA) is a key consideration in our experiments. Based on the recommendations from the supplier, CA powder was stored in a refrigerator at 3–5 °C prior to use. During the experimental process, preconditioning for the RCPT and after the tests, all specimens were maintained at consistent laboratory condition. These controlled conditions helped to preserve the stability and functionality of the enzyme during the testing phase. It should be considered that investigating the homogeneity of carbonic anhydrase (CA) activity in concrete is a complex challenge, because of the inherent heterogeneity of the concrete itself. Additionally, a trace amount of CA was added in our study to 12 L of concrete during its preparation in each batch. As a result, it is challenging to assess the CA distribution and activity on a large scale directly.

### 2.2 Mix Proportions and Preparation of Specimens

The main components in preparing concrete mixtures are Portland cement (CEM I), aggregates, and deionized (DI) water. Three series of concrete specimens were made. Control is a group of specimens without CA and two groups of specimens containing low and high dosages of CA. The dosages of CA are defined as 5 and 10 times, named CA-5X and CA-10X, respectively. One dosage is 0.29 mg of CA solved in 1 mL of DI water per 1 kg cement. For 5 and 10 times dosages (CA-5X and CA-10X), the amounts of required CA and DI water were multiplied by 5 and 10 per 1 kg cement, respectively. Water-to-cement ratios were similar for all mixtures at 0.45. Figure 2 shows aggregate size distributions. The total aggregate-to-cement ratio was 2.4 (Table 2).

**Figure 2:**
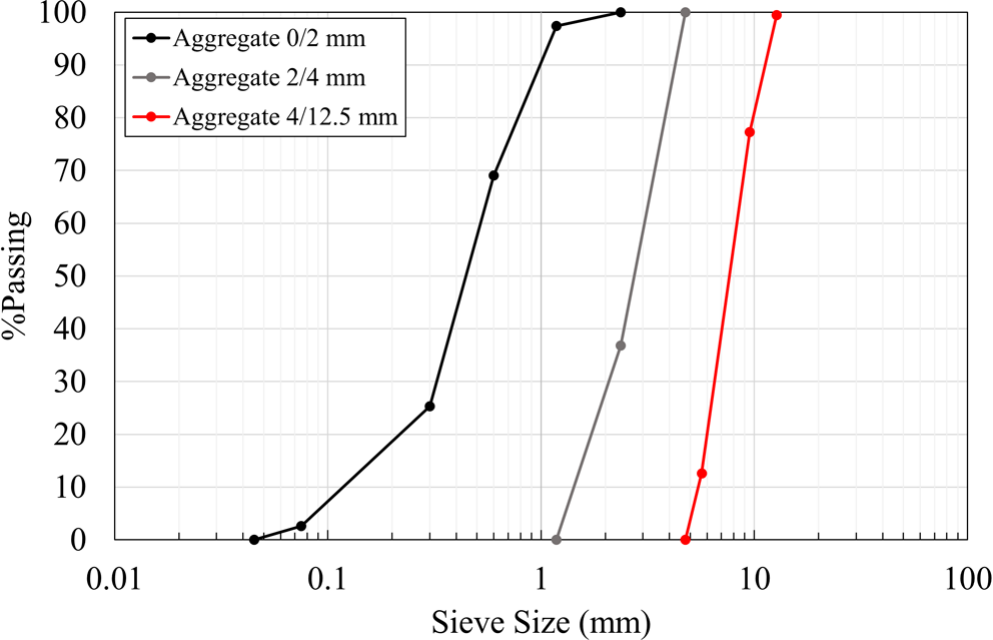
Aggregate size distribution of sand grading between 0 and 2 mm and from 2 to 4 mm and crushed coarsely with a grading between 4 and 12.5 mm.

**Table 2:** Mix Designs of Concrete without CA (Control), Containing 5 Times Dosages of CA (CA-5X), and Containing 10 Times Dosages of CA (CA-10X)

ratio	control	CA-5X	CA-10X
water to cement	0.45	0.45	0.45
aggregate 0/2 mm to cement	0.92	0.92	0.92
aggregate 2/4 mm to cement	0.34	0.34	0.34
aggregate 6/12.5 mm to cement	1.26	1.26	1.26
carbonic anhydrase (mg) per 1 kg cement		5 × 0.29	10 × 0.29
slump (cm)	24.5	22.5	21
air content (%)	1.00	0.85	0.90
compressive strength at 28 days (MPa)	29.5	34.3	35.7

The compositions of concrete mixtures are shown in Table 2, as are their fresh and hardened properties. The information on the effect of CA and dosage on the properties of concrete is also presented. To determine the air content and slump flow as fresh properties of concrete, standardized procedures provided by ASTM C231/C231M-17a^34^ and ASTM C143/C143M-12 ^35^ were followed. The air content and slump flow reduced slightly with using CA compared to the control. This reduction may be due to the presence of calcite crystals and reduced porosity of concrete.^36^

To study the compressive strength, as a fundamental mechanical property of concrete, cylindrical molds with 10 cm diameter and 20 cm height were filled and tested at the curing age of 28 days based on ASTM C39.^37^ RCPT was performed on uncracked concrete at two ages: 14 and 28 days. Before the RCPT preconditioning was applied, the cylindrical concrete was cut into three cylindrical disks with a height of 5 cm, and the middle disk was chosen from each cut cylinder for the RCPT. The RCPT was performed only at the 28-day age for cracked concrete disks. Two sizes of molds were used to evaluate the durability properties of concrete. Cylindrical molds had 10 cm diameter and 20 cm height for uncracked concrete, and disk molds had 10 cm diameter and 5 cm thickness for cracked concrete specimens.

For casting concrete, aggregates and cement were mixed together for 1 min, then water was added to the mix, while cement and aggregates were mixed for 30 s. After the entire water was poured, they were mixed for two more minutes. The CA solution was mixed with water for the concrete mix containing CA, and then, the water was added to the concrete mix at this time. Slump and air-entrained content of fresh concrete were tested for its fresh properties. After casting concrete and filling the molds, they were stored in a room with a controlled relative humidity and temperature for 24 h. After 24 h, the concrete specimens were removed from the mold and cured in the fog chamber with 95 ± 5% relative humidity and temperature 23 ± 2 °C until the tests were performed.

### 2.3 Crack Formation and Crack Sealing

Corrosion-induced cracking typically originates internally, as corrosion products expand and induce tensile stresses that propagate cracks outward. Conversely, external cracks form at the surface owing to tensile stresses and extend inward toward the reinforced concrete. Sealing these surface cracks is critical for minimizing the chloride ingress and delaying the onset of corrosion at the rebar surface.^22^

Disk molds with dimensions of 10 cm and thickness of 5 cm were filled with fresh concrete. Then, a Teflon tape with a thickness of 0.5 mm, a length of 2 cm, and a depth of 1.5 cm was placed in the middle of the concrete disk (Figure 3). After concrete disk specimens were removed from the molds, the tape was pulled out of the specimens. Then, they were cured in a fog chamber for 28 days.

**Figure 3:**
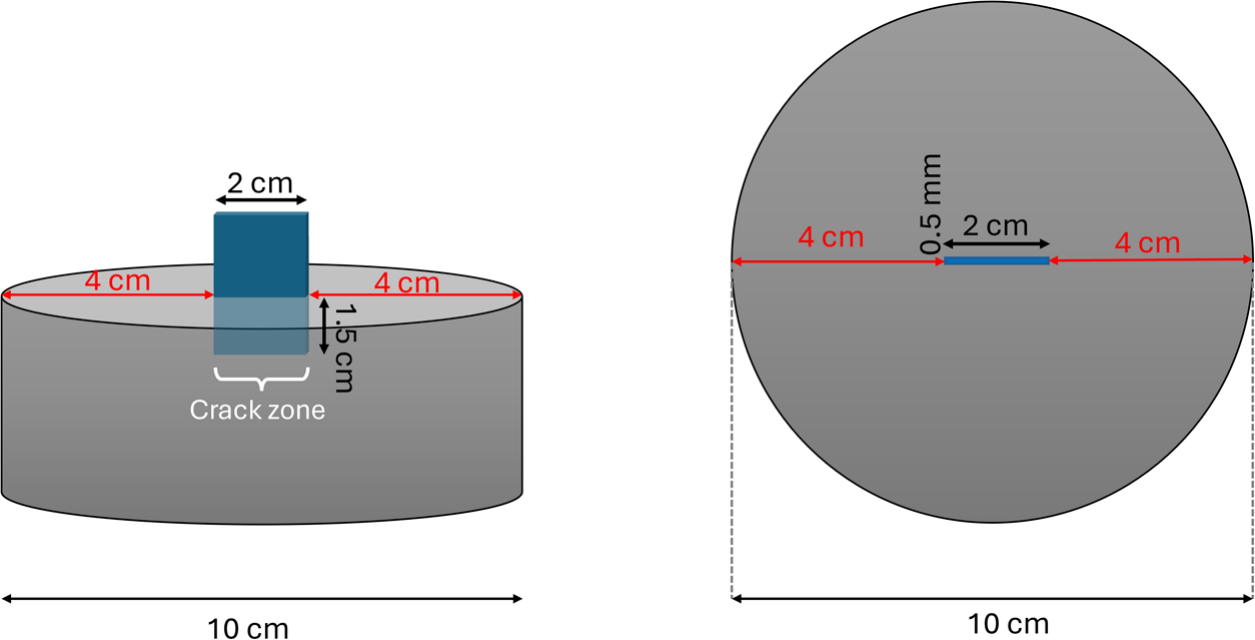
Geometry and location of the tape placed in the fresh concrete disk specimen during casting to introduce a notch, left: side view, right: top view.

The optimal performance of the enzyme occurs at pH 8, whereas concrete typically exhibits a higher pH (approximately 12). Although the efficiency of CA is lower at high pH, we initially investigated whether the enzyme remains active through the effect of its presence on accelerating the sealing of cracks by comparing it to a cracked control sample that did not contain any enzyme. We explored the use of carbonic anhydrase (CA) as a sealing agent to accelerate the reaction between a calcium source and CO_2_, leading to the precipitation of CaCO_3_. Therefore, 22 g of calcium formate was dissolved in 50 mL of deionized (DI) water, and 5 mL of this solution was sprayed onto the crack zone of the cracked control and cracked CA-5X samples. The cracked control specimen was then exposed to a constant CO_2_ gas flow in air for 6 h, with the CO_2_ concentration in the gas phase under standard conditions (24 °C and 1 atm) of 0.0415 mol/L. The cracked CA-5X sample was placed in ambient air, and 5 mL of calcium solution was sprayed on the crack zone during the same 6 h period of sealing of the cracked control specimen. Based on our initial investigation into the crack sealing of concrete with and without enzymes (Section 3.1), and considering previous studies^19,36^ demonstrated that CA-5X produced the most optimal dosage to improve durability parameters, we aimed to run the RCPT at the same age for all samples, including uncracked, unsealed, and sealed cracked concrete, to facilitate a direct comparison. In the second phase, we compared the effect of cracks before and after sealing on the chloride penetration of CA-5X. Specifically, we examined chloride penetration in areas far from the crack zone to assess how the distance from the crack influences chloride penetration before and after crack sealing. These comparisons provide insights into the role of cracks in chloride penetration and the potential of CA-5X to mitigate this issue through sealing. After curing and before starting the sealing process, the crack width was measured at 0.25, 0.5, and 0.75 of the crack length. The average of three measurements was reported as the crack width.

To seal the crack, 22 g of calcium formate was dissolved in 50 mL of deionized (DI) water. Subsequently, 5 mL of this solution was sprayed in the crack zone to seal the cracks. It should be noted that carbonic anhydrase was not added to this solution, and it was only added to the concrete mix during casting concrete. The cracked specimen was then exposed to constant CO_2_ gas in the air for 6 h. The concentration of CO_2_ in the gas phase at a standard temperature of 24 °C and pressure of 1 atm is 0.0415 mol/L. During the sealing procedure, the progression of crack sealing was monitored at the same points that, previously, the crack widths were measured. As shown in eq 3, the percentage of the sealing efficiency (SE%) was calculated based on the average crack sizes before sealing, after 3 h of sealing, and at the end of the sealing process. SCW represents the average crack width of a specimen before sealing (*t*_0_) and after *t* hours of sealing time.

Because we used the addition of calcium solution and a constant supply of CO_2_ gas, it qualifies as autonomous self-sealing concrete.^20^ This is because the sealing process is triggered automatically when the necessary external agents (CO_2_ and calcium) are introduced without further human intervention, and carbonic anhydrase accelerates the reaction to precipitate calcium carbonate in the crack zone.

3 $$\mathrm{SE}\%=\frac{\mathrm{SC}W_{t_{0}}-\mathrm{SC}W_{t}}{\mathrm{SC}W_{t_{0}}}\times 100$$

### 2.4 Rapid Chloride Penetration Test (RCPT)

We monitored the chloride penetration depth and chloride migration coefficient of concrete specimens by a non-steady-state chloride migration test method, hereafter called simply RCPT, according to NT BUILD 492 ^12^ on concrete specimens listed in Table 3. Based on our study on the effect of CA dosage on the chloride penetration of uncracked concrete (Section 3.2), investigation into the crack sealing of concrete with and without enzymes (Section 3.1) and considering previous studies that demonstrated that CA-5X produced the best results in improving durability parameters,^19,36^ we aimed to run the RCPT at the same age for all samples, including uncracked, unsealed, and sealed cracked concrete, to facilitate a direct comparison. To achieve this, we used CA-5X samples that underwent crack sealing by CO_2_ bubbling while simultaneously spraying a calcium solution for 6 h to thoroughly seal the cracks thoroughly. This approach allowed us to perform RCPT on these samples at the same age (28 days) as the other concrete samples, ensuring consistent conditions for comparison and allowing us to assess the effect of crack sealing on chloride penetration resistance.

**Table 3:** Framework of RCPTs on Concrete Specimens

age (day)	crack condition	type	repetition
14	uncracked	control	3
		CA-5X	3
		CA-10X	3
28	uncracked	control	7
		CA-5X	7
		CA-10X	7
	unsealed cracked	CA-5X	3
	sealed cracked	CA-5X	3

After the cylinder was cut into a disk, the resulting disk had two exposed surfaces. RCPT was conducted on this disk, with chloride attack applied to the upper and lower surfaces for uncracked specimens at 28 days. Specifically, four and three specimens were tested on the upper and lower surfaces, respectively. For uncracked specimens at the age of 14 days, the lower surface was only exposed. Since larger aggregates tend to settle at the lower part of the specimen during the mold-filling process, the lower surface of the disk specimens contains a higher concentration of larger aggregates. Therefore, we have chosen these specimens for comparison to minimize the effect of aggregate size distribution and consider the worst-case scenario, specifically in the estimation of the service life of concrete on the result. After the RCPT was run on uncracked concrete without and with different dosages of CA at 14 and 28 days of age, concrete containing a lower CA (CA-5X) was considered to study the effect of crack sealing on chloride penetration.

NT BUILD 492 standard provides the guidelines for the RCPT method only for uncracked concrete. We designed the geometry of a crack for a concrete disk specimen to follow precisely the standard and kept the test condition the same for uncracked and cracked concrete specimens. This approach results in a comparable evaluation of the effect of using different dosages of CA, the presence of cracks, and the sealing of the cracks on the chloride penetration of concrete based on the standard. In addition, the effect of the crack presence on the chloride penetration through the crack faces and tip can be observed.

A preconditioning should be applied to concrete specimens before the start of an RCPT. Disk specimens were vacuumed with air for 3 h. Then, while the vacuum pump was still running for one more hour, DI water was slowly filled into the desiccator to immerse all of the specimens completely. Then, the vacuum was turned off, and specimens were kept in DI water for 18 ± 2 h.

After the sample was vacuumed, the concrete disk specimens were placed in the RCPT cell, as shown in Figure 4. First, the concrete disk was placed in the spacer, and then two impermeable rubbers were attached to the two ends of the specimen. High vacuum grease was spread anywhere the spacer and impermeable rubbers were in contact with the concrete specimen to seal it thoroughly. The saturated disk specimen placed in the middle of the spacer was positioned in the cell, and again, high vacuum grease was used to ensure there would not be any leaching around the placed disk specimen in the cell. Four steel bolts were used to fix the cell setup at each corner of the cell. When the cell setup was ready, both cell chambers were filled with DI water to check the water-tightness of the cell, and no leaching occurred during the test. When we were assured that the specimen was sealed well, DI water was removed, and each cell chamber was filled with the related solutions. The NaCl chamber of the cell was filled up with a 10% NaCl solution, and the NaOH chamber was filled with 0.3 M NaOH solution. The initial current through each specimen was recorded with a preset voltage of 30 V to find the appropriate voltage and duration. Then, they were chosen based on the table provided by NT BUILD 492.^12^

**Figure 4:**
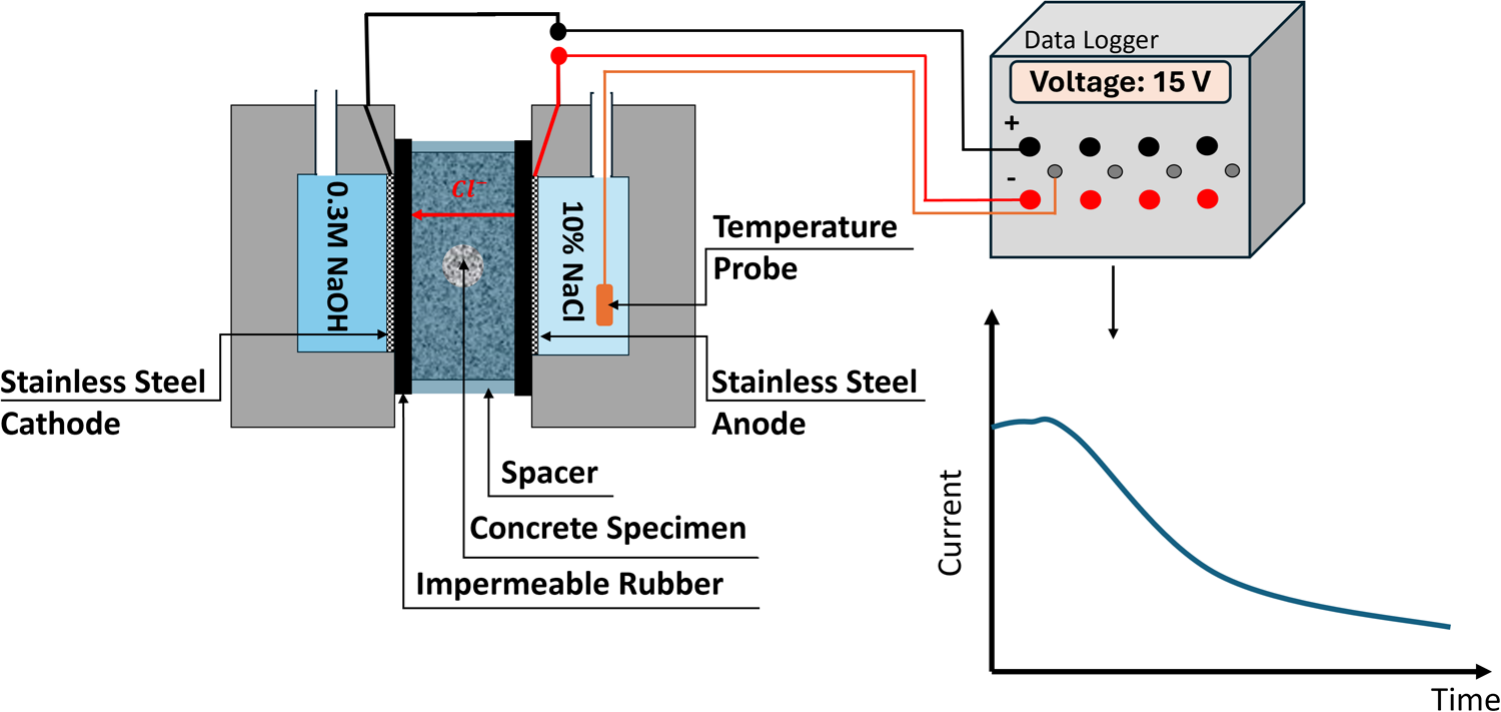
Schematic of the RCPT setup. The actual applied voltage, initial and final currents, initial and final temperatures, and elapsed time are digitally recorded.

At the end of the experiment, the cell was disconnected from the power supply and the specimen was removed from the cell. Then, the disk specimen was split immediately using the Brazilian test, and 0.1 M silver nitrate (AgNO_3_) solution was sprayed on both halves of a split disk. The formation of AgCl turns the concrete area containing a high chloride concentration white. Then, the chloride penetration depth was measured at each 10 mm interval from the center to 10 mm from the edges at each half of the disk with a caliper accuracy of 1 μm. If a large aggregate blocked any penetration front at the measured point, then the measurement point was moved to the nearest point where no significant blocking was observed.

The average chloride penetration depth was calculated among 14 measurements in total for one concrete disk (7 measurements from each half). This average value was used to calculate the rapid chloride migration coefficient (*D*_RCM_). *D*_RCM_ can be derived from the diffusion equation (eq 4) for the non-steady-state test condition as^12^

4 $$D_{RCM}=\frac{0.0239\cdot \left(273+T\right)\cdot L}{\left(U-2\right)\cdot t_{d}}\left[\chi_{d}-0.0238\sqrt{\frac{\left(273+T\right)\cdot L\cdot \chi_{d}}{U-2}}\right]$$

where *D*_RCM_ is the rapid chloride migration coefficient (10^–12^ m^2^/s); *U* is the value of the applied voltage during the test (V); *T* is the mean value of the initial and final temperatures in the anolyte solution °C; *L* is the thickness of the disk specimen (mm); χ_d_ is the mean value of 14 measurements of chloride penetration depths of the disk specimen (mm); and *t*_d_ is the duration of the test (h).

### 2.5 Material Characterizations

Microanalysis of thin sections from the uncracked tested specimen was conducted to verify the presence and distribution of Friedel’s salt in the area containing high chloride concentration. The mechanical polishing can lead to the loss of surface layers or modify the surface properties, potentially washing out or masking the presence of phases such as Friedel’s salt.^38^ Using unpolished samples helps preserve the original microstructure and ensures a more accurate examination of the material as it exists in its natural state without the risk of contaminating or losing critical phases during preparation. Thin samples from a tested concrete disk with a size of 3 mm × 3 mm × 1 mm were removed at two locations, from the area accessed by chloride and the area that chloride did not reach at the end of the test (19–21 mm depth). Scanning electron microscopy (SEM) and energy-dispersive X-ray spectroscopy (EDX) were used to examine the surface morphology and phase composition. X-ray diffraction (XRD) was used to characterize the crystal structures and to observe Friedel’s salt in a concrete powder sample collected from the area containing chloride. The results were compared with the area that chloride did not reach during the RCPT.

### 2.6 Inductively Coupled Plasma–Mass Spectrometry (ICP-MS)

The colorimetric methods are fast and widely used. However, these methods are qualitative, and the chloride concentration at the changing color boundary is varied.^39^ Hence, we measured the chloride concentrations at different depths of the specimens, with and without the crack, at 28 days by the inductively coupled plasma–mass spectrometry (ICP-MS) technique. We needed to grind the specimen and measure the mass of the powder to measure the chloride penetration in the tested specimens at different depths. Immediately after reading the chloride penetration, two halves of the specimen were reassembled and held together using tape. Then, a concrete grinder was used to grind every 2 mm layer, and the powder was collected at each level. To improve the accuracy, the area near the opening surface, where AgNO_3_ was sprayed, and a 1 cm layer from the lateral surface of the tested disk specimen were not included. Grinding started from the specimen’s exposed surface to a maximum of 21 mm depth inside the disks. However, the first layer was discarded and not used to calculate the chloride content. Therefore, the rest of the 9 powder samples representing each layer depth were collected for each tested disk specimen.

After grinding and collecting the concrete powder at each 2 mm depth, 1 g from each layer was dissolved in 50 mL of DI water. Then, it was placed on a hot plate at 60–80 °C for 1 day to ensure that the entire chloride was dissolved in water. This temperature was chosen to ensure efficient preparation of the solution while minimizing unwanted reactions after removing the solution from the hot plate. The solution was filtered and brought up to 50 mL. As it was assumed that the chloride concentration in initial levels would be too high, 0.5 mL of prepared solutions from the first (0–2 mm) and second (2–4 mm) layers were dissolved in 50 mL of DI water as a final solution. 1 mL of prepared solutions from the third (4–6 mm), fourth (6–8 mm), and fifth (8–10 mm) layers were dissolved in 50 mL of DI water accordingly. As the chloride concentration decreased in depth, 5 mL of remaining solutions from deeper layers were dissolved in 50 mL of DI water. These 9 solutions were placed in the ICP-MS machine to measure the percentage of chloride in concrete powder.

## 3 Results and Discussion

### 3.1 Crack Sealing

Our study aimed initially to address the sealing of wide cracks as early as possible, which poses a greater challenge, particularly when aggressive agents can reach the rebar. We explored the use of carbonic anhydrase (CA) as a sealing agent to speed up the reaction between the calcium source and CO_2_ leading to the precipitation of CaCO_3_. So, 22 g of calcium formate was dissolved in 50 mL of deionized (DI) water, and 5 mL of this solution was sprayed on the crack zone. The cracked control specimen was then exposed to a constant CO_2_ gas flow in the air for 6 hours, with the CO_2_ concentration in the gas phase at standard conditions (24 °C and 1 atm) being 0.0415 mol/L. No significant crystal growth was observed in the crack zone of the control sample after 6 h of sealing (Figure 5a). Interestingly, more crystal growth was observed in CA-5X sample that was placed in ambient air and 5 mL of calcium solution was sprayed on the crack zone during the same 6 h period of sealing the cracked control specimen (Figure 5b). This result is remarkable as the CA-5X sample, though not directly exposed to the CO_2_ gas pump, exhibited more crystal formation compared to that of the control. Based on our initial investigation into the crack sealing of concrete with and without enzymes, influence of CA dosage on the chloride penetration of uncracked concrete through RCPT, which is discussed in Section 3.2, and considering previous studies that demonstrated CA-5X produced the best results in improving durability parameters,^19,36^ we aimed to run the RCPT at the same age for all samples, including Uncracked, unsealed and sealed cracked concrete, to facilitate a direct comparison. To achieve this, we used CA-5X samples that underwent crack sealing by CO_2_ bubbling while simultaneously spraying a calcium solution over 6 h to fully seal the crack at the surface. This approach allowed us to perform the RCPT on these samples at the same age (28 days) as the other concrete samples, ensuring consistent conditions for comparison and allowing us to assess the effect of crack sealing on chloride penetration resistance.

**Figure 5:**
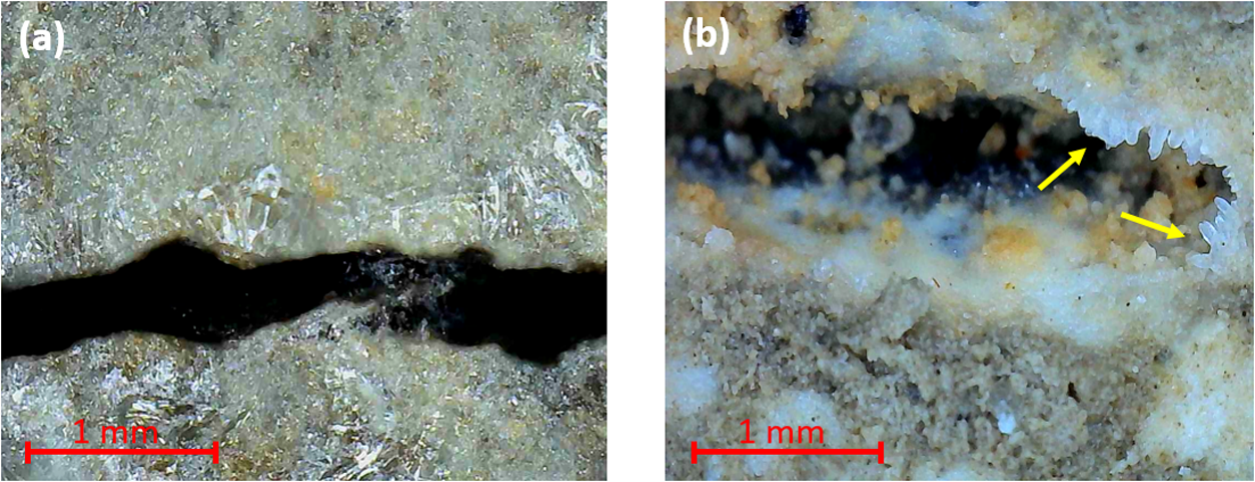
Progression of crack sealing over a 6 h exposure period in (a) a cracked control sample under CO_2_ bubbling, showing no crystal growth, and (b) a cracked CA-5X sample under ambient air conditions, where crystal growth is indicated by yellow arrows.

The progression of crack sealing was observed when the sealing agent was applied over 6 h. As shown in Figure 6, crack sealing was observed through a digital optical microscope at the surface of the specimen. The crack width was measured before the sealing started (Figure 6a), after 3 h of sealing (Figure 6b), and at the end of sealing time (Figure 6c). The average crack width before sealing was 500 μm; in the middle of sealing, it was around 225 μm, on average. So, the Sealing Efficiency (SE) of 55% was observed after a 3 h progression of crack sealing. At the end of the crack sealing regime, no opening was found at the crack surface, and it was fully sealed. Therefore, sealing the crack showed the capability of 100% E when using the 5X enzyme dosage in the enzymatic concrete specimens (CA-5X) in a short period.

**Figure 6:**
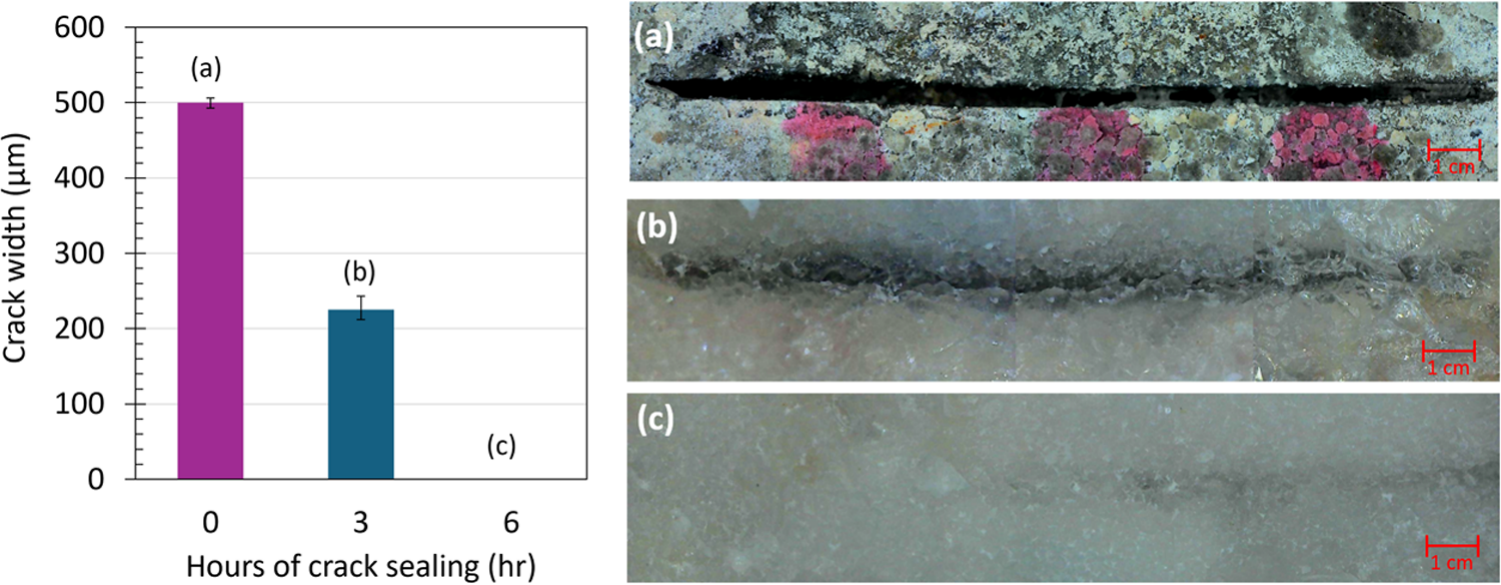
Closure of a wide crack (0.5 mm width) at the surface of the CA-5X concrete specimen (a) before spraying the calcium solution; (b) 3 h after spraying the calcium solution, an average of 55% sealing efficiency was observed; and (c) 6 h after spraying the calcium solution, 100% sealing efficiency was observed.

### 3.2 Influence of CA Dosage on the Chloride Penetration of Uncracked Concrete

The adjusted voltage of 15 V was applied on all specimens for 24 h, as recommended by the standard^12^ to keep similar test conditions and be able to compare the results among different series. During the test, the temperature was measured frequently and did not fluctuate more than 2 °C, indicating that the voltage and duration were appropriate for the specimens. The average temperature during the test is 24 °C. The color change boundary method was used to assess the development of chloride penetration by spraying silver nitrate, and the effect of two different dosages of CA on *D*_RCM_ was examined at two ages of 14 and 28 days. Results are presented in Figures 7 and 8.

**Figure 7:**
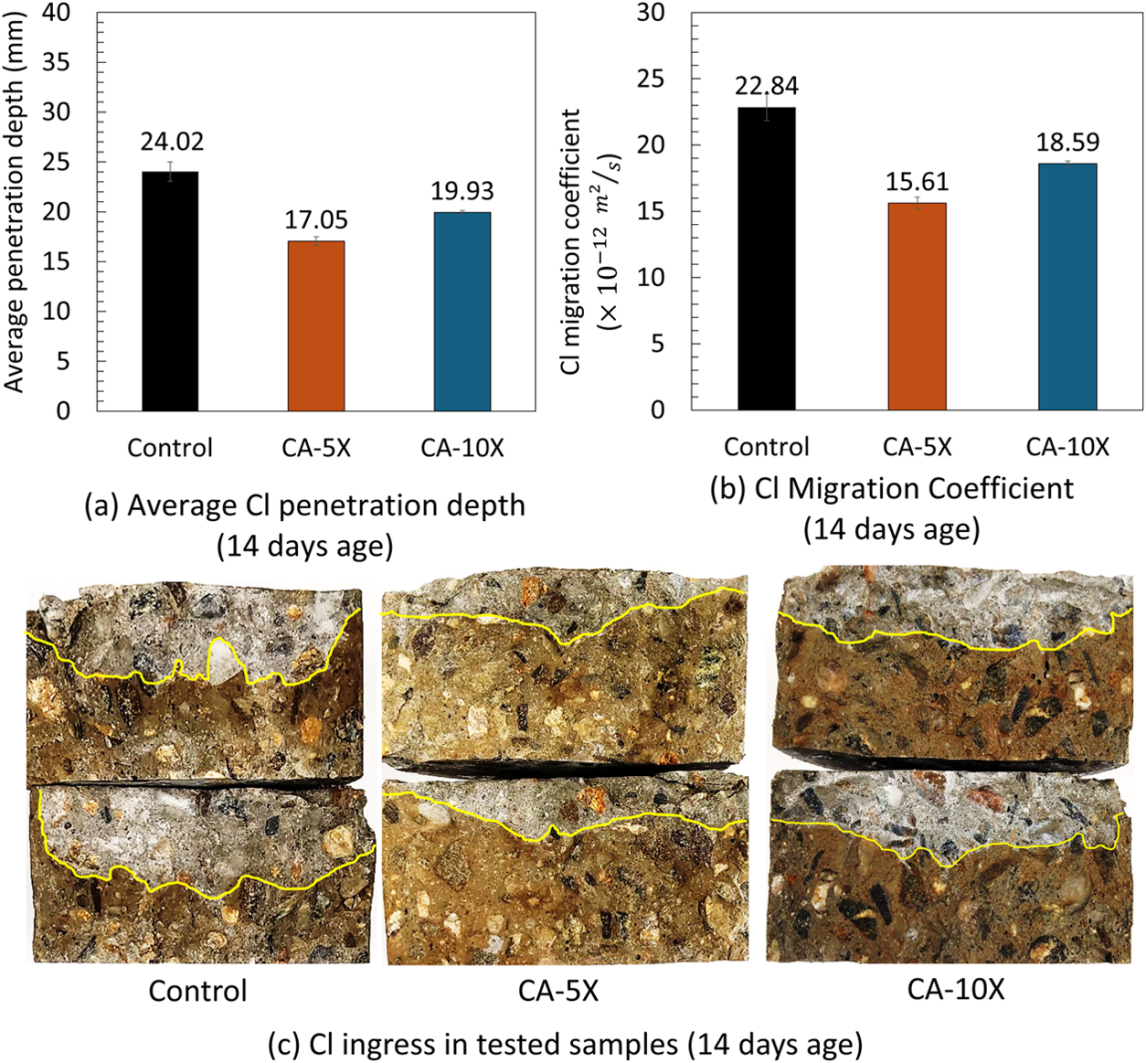
(a) Average chloride penetration depths of three types of uncracked concrete specimen at the age of 14 days, (b) average chloride migration coefficient of three types of uncracked concrete at the age of 14 days, and (c) an example of Cl ingress in tested specimens at 14 days age after spraying silver nitrate; the white area shows the presence of Cl.

**Figure 8:**
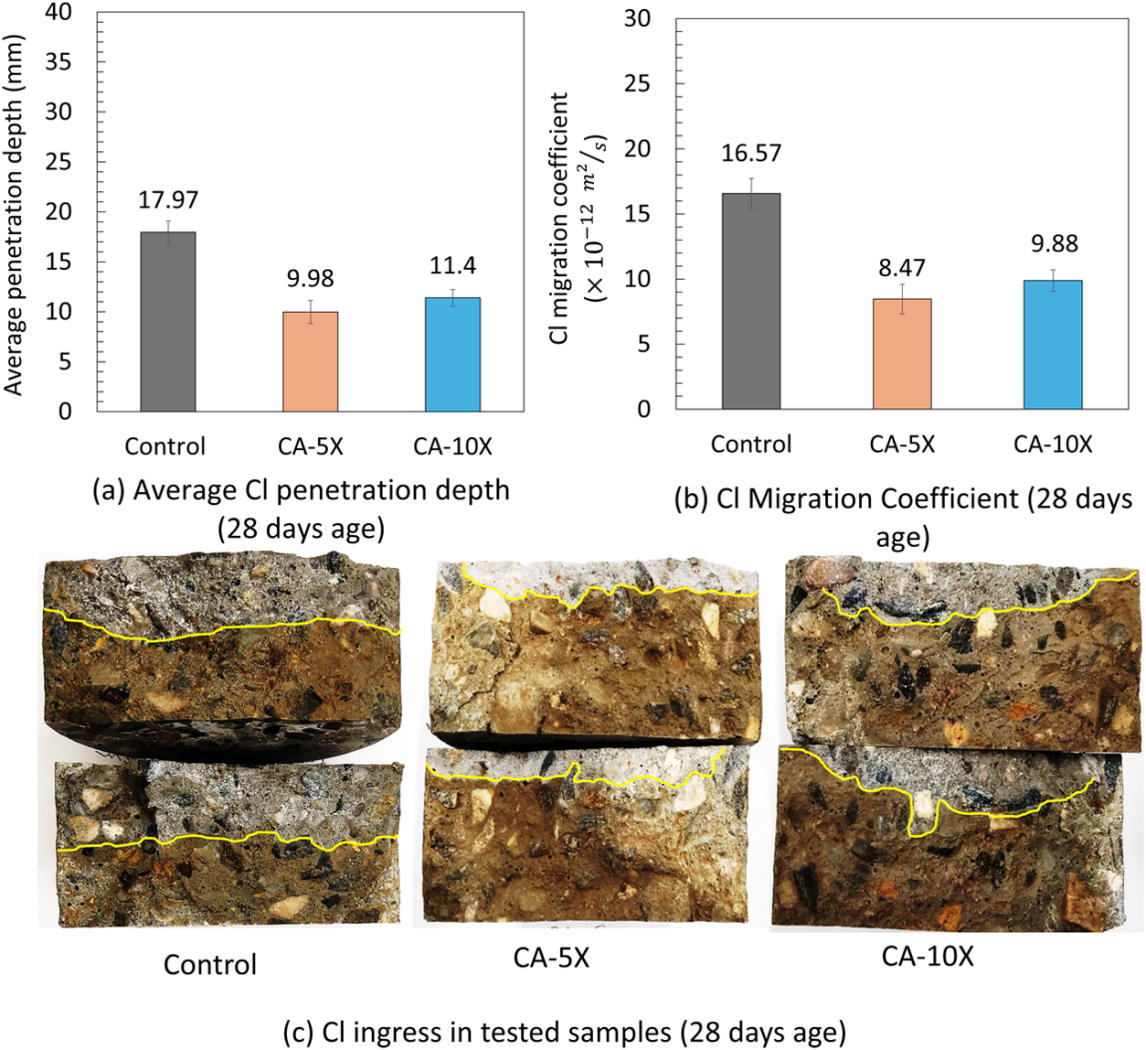
(a) Average chloride penetration of three types of uncracked concrete at the age of 28 days. The average chloride penetration of uncracked control, CA-5X, and CA-10X specimens that lower surface exposed to the chloride solution is 17.87, 10.38, and 11.69 mm, respectively. (b) Average chloride migration coefficient of three types of uncracked concrete at the age of 28 days. (c) Example of Cl ingress in tested specimens at 14 days age after spraying silver nitrate; the white area shows the presence of Cl.

Seven specimens were tested at 28 days of age. Four specimens had their upper surface exposed to chloride solution during RCPT, while three had their lower surface exposed with all specimens uncracked. The results demonstrated that chloride penetration was reduced by 44.5% and 36.6% in the specimens containing CA-5X and CA-10X, respectively, compared to the control specimens, as shown in Figure 8a,8c. Moreover, under constant testing conditions, the chloride migration coefficient decreased by 48.9% in the CA-5X specimens and by 40.1% in the CA-10X specimens when compared with the control specimen (Figure 8b). The average chloride penetration for the uncracked control, CA-5X, and CA-10X specimens, with their lower surface exposed to the chloride solution, was 17.87, 10.38, and 11.69 mm, respectively. This difference can be attributed to the casting process, where larger aggregates were placed in the lower part of the concrete, while the upper part may have been denser. As a result, chloride penetration was slightly higher in the lower surface-exposed specimens compared to those with the upper surface exposed to chloride attack. Therefore, the specimens with the lower surface exposed to chloride solution are considered for comparison and calculation of service life. The chloride ingress decreases by increasing the curing time from 14 days to 28 days in control, CA-5X, and CA-10X specimens by 25.6%, 39.1%, and 41.3%, respectively. This trend indicates that curing age influences the resistance of Portland cement to chloride ingress as it is almost completely hydrated after 28 days of moist condition. The control series illustrated significantly higher chloride ingress than the series containing CA, and no significant impact was observed on the chloride ingress of concrete containing CA at both ages. The results clearly show that using a trace amount of CA in concrete reduced the chloride penetration of concrete considerably. For instance, at 14 days, CA-5X reduces the chloride penetration by 29%, and by increasing the curing time from 14 days to 28 days, the chloride penetration further decreased to 41.9%. Decreasing the chloride permeability of CA-5X and CA-10X compared to the control is due to the precipitation of the crystals in the flaws and pores of the concrete, which is aligned with the mercury intrusion porosimetry (MIP) results demonstrated that the CA-5X sample exhibited significantly lower permeability and higher tortuosity compared to the control, indicating enhanced matrix densification and reduced pore connectivity.^36^ Increasing the dosage of CA from 5 to 10 times shows a positive impact on the reduction of chloride ingress by 17% and 34.5% in comparison with the controls at 14 and 28 days of age, respectively. This trend suggests a potential saturation effect that could limit the effectiveness of CA at a higher dosage (CA-10X). At high concentrations, CA may precipitate out of the solution, resulting in a lack of homogeneity. (Figures 7a,7c and 8a,8c). This is aligned with the advantage of using CA to reduce the water permeability of concrete and increase its service life.^19^

The effect of different dosages of CA on chloride migration coefficient at 14- and 28-day ages is presented in Figures 7b and 8b. Based on NTBUILD 492,^12^ the two most outlier penetration depths were removed from each half of a broken disk specimen, and the average chloride penetration depth was then calculated among 14 measured points for each disk specimen. The second Fick’s law was then used to calculate the *D*_RCM_. Comparing the coefficient of specimens that lower surface exposed to the chloride solution showed that (*D*_RCM_) of control, CA-5X, and CA-10X decreased 28, 43, and 45%, respectively. The results showed that longer curing contributed to the densification of the concrete matrix by completing the cement hydration process, aiding the lower permeability of concrete. The (*D*_RCM_) of control is always more than the CA specimens. It is 50% larger than CA-5X and 20% larger than CA-10X at 14 days. With increasing curing time, the differences are more pronounced due to the simultaneous hydration of Portland cement. At the 28-day age of specimens, (*D*_RCM_) of control is 80 and 60% higher than that of CA-5X and CA-10X, respectively (Figures 7b and 8b). A considerable reduction of the diffusion coefficient in CA-5X and CA-10X showed a significant effect of CA on extending the service life of concrete, which is discussed in Section 3.6.

The current passing through the concrete disk was the other parameter that was monitored at the start and end times of the RCPT after adjusting the voltage. Based on Ohm’s law (*V* = *I* · *R*), the current is directly proportional to voltage but inversely proportional to resistance. As the voltage was constant over the test period, the changes in actual current showed changes in concrete resistivity during the test. For uncracked concrete, the actual currents of CA-5X at the initial and final test times were not higher than the control at 14-day age (Figure 9a). This consistency in current clearly showed that the resistivity of CA-5X had been higher than that of the control during the test, which aligns well with the lower chloride penetration of CA-5X compared with the control.

**Figure 9:**
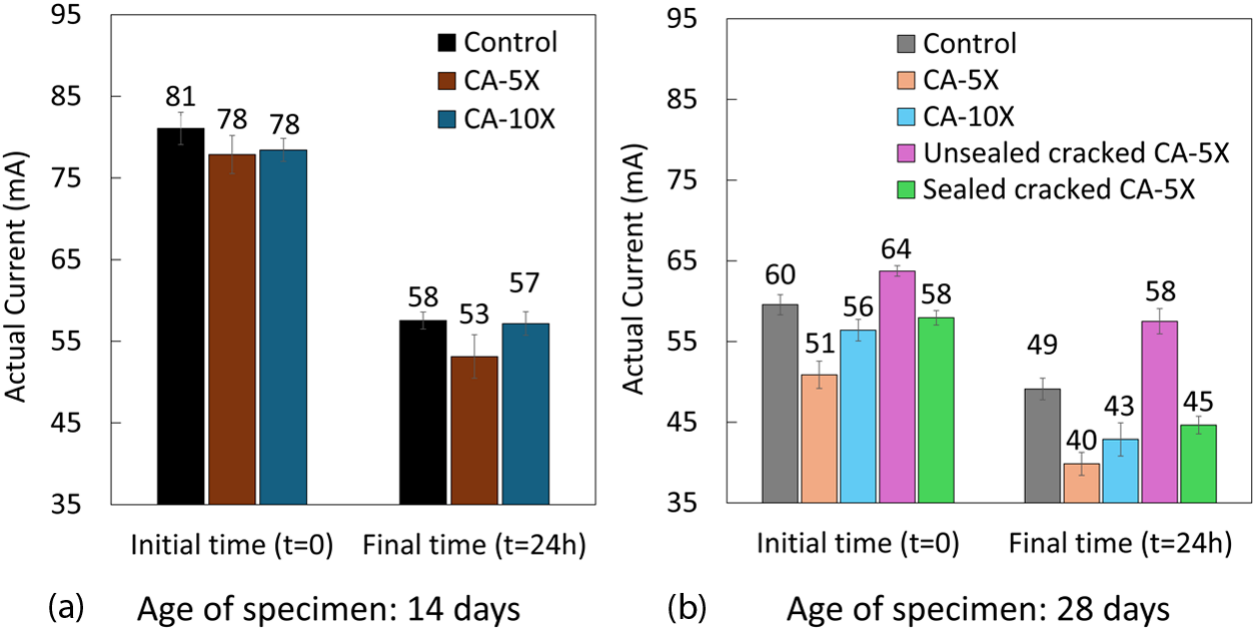
Measured currents (mA) at the initial and final stages for (a) uncracked concrete at 14 days of age and (b) uncracked, unsealed cracked, and sealed cracked concrete specimens at 28 days. The error bars represent the standard deviation, and each experiment is repeated three times.

We believe that crystal growth in concrete pores of specimens containing CA and simultaneous cement hydration causes the measured currents in CA-5X and CA-10X to be much lower than the control at 28 days at the initial and final testing times. For example, the measured current in control, CA-5X, and CA-10X were 60, 51, and 56 mA at the start time of the test and 49, 40, and 43 mA at the end of the test, which showed that the resistivity increased at the end of the test for all series and the rate of increasing the resistivity is 0.22 for control, 0.28 for CA-5X, and 0.3 for CA-10X, respectively. It might contribute to the higher dosage of CA showing a higher rate of resistivity growth (Figure 9b). This aspect is discussed extensively in Section 3.4.

### 3.3 Influence of Cracking and Sealing on the Chloride Penetration of Concrete

As the chloride penetration of uncracked CA-5X specimens was the lowest and we aimed to perform RCPT on samples at the same age (28 days), ensuring consistent conditions for comparison and allowing us to assess the effect of crack sealing on chloride penetration resistance, CA-5X was chosen to evaluate the effect of crack sealing on the chloride penetration of cracked CA-5X specimens. To compare the impact of crack presence and crack sealing on the chloride penetration of concrete, all test conditions were kept the same as those of uncracked CA-5X specimens. In cracking conditions, unsealed or sealed was the only variable. The voltage of 15 V was applied on unsealed and sealed cracked concrete specimens for 24 h. The average temperature is 24 °C during the test, and no difference more than 2 °C was observed, which showed the voltage and duration were appropriate for these specimens.

The actual current at the initial and final times was recorded during the test. As shown in Figure 9b, in the initial hours of the RCPT test, the current for the “Unsealed Cracked CA-5X” specimens increased because of enhanced conductivity, owing to the presence of voids and flaws in the concrete microstructure. However, after a certain period, the current begins to decrease. This could be attributed to the precipitation of crystal ions in the nano- and micropores, which may have partially blocked the pathways, reducing the current flow. Further studies are required to analyze the microstructural changes in more detail to determine whether any crack sealing occurred during the test fully. Sealing the crack with the 100% SE at its surface reduced the actual current to a comparable level with the control specimen. For example, the initial actual current was 58 mA for sealed concrete, and at the end of the test, the final value was 45 mA. This current drop confirms that using a trace amount of CA in concrete can efficiently seal the crack, increase the concrete resistivity due to the activation of CA during the crack sealing, and speed up the crystal growth in concrete, resulting in denser concrete.

Similar to the uncracked concrete specimens, 1 cm of the lateral cut surface of each half of a tested specimen was not considered for the chloride penetration measurement, as shown in Figure 10a,10b. In addition to the total chloride penetration in cracked concrete, chloride penetration is studied in two separated zones: the crack zone (shown with a white dashed square) to assess the pure effect of crack presence and crack sealing on chloride ingress and the uncracked zone (0.5 cm far from each side of the crack) to study the impact of crack presence and sealing on the chloride ingress in the area far from the crack (Figure 10b).

**Figure 10:**
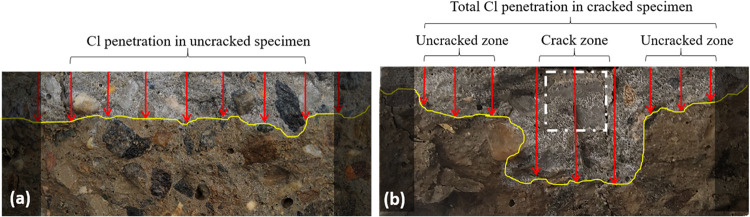
Average chloride penetration depth through the exposed surface of the concrete specimen after spraying the silver nitrate and removing 1 cm from the lateral surface in (a) uncracked specimen with 9 measurements with the spacing of 1 cm in the uncracked specimen and in (b) cracked specimen with 3 measurements at the crack zone and 6 measurements from the right and left of the crack zone with a spacing of 1 cm (uncracked zone).

Figure 11 shows the RCPT results of cracked concrete before and after sealing. In unsealed cracked concrete, the presence of a crack leads to loss of resistivity to chloride transport and rapid penetration of chloride through the crack, which is around 2.7 times higher than the uncracked zone (Figure 11a). It must be noted that a comparison of the chloride penetration depths and migration coefficients of the uncracked zone of cracked concrete (Figure 11) with that of uncracked concrete (Figure 8a,8b) does not show any significant difference in the chloride ingress at this zone. Sealing the crack clearly shows the advantage of using CA in concrete to recover its resistivity among the chloride ingress at the crack zone. Sealing the crack reduced the chloride penetration at the crack zone up to 55%, and the migration coefficient at both uncracked and crack zones of sealed specimens (Figure 11) is similar to the control specimen without crack and CA-5X specimens at 28 days (Figure 8).

**Figure 11:**
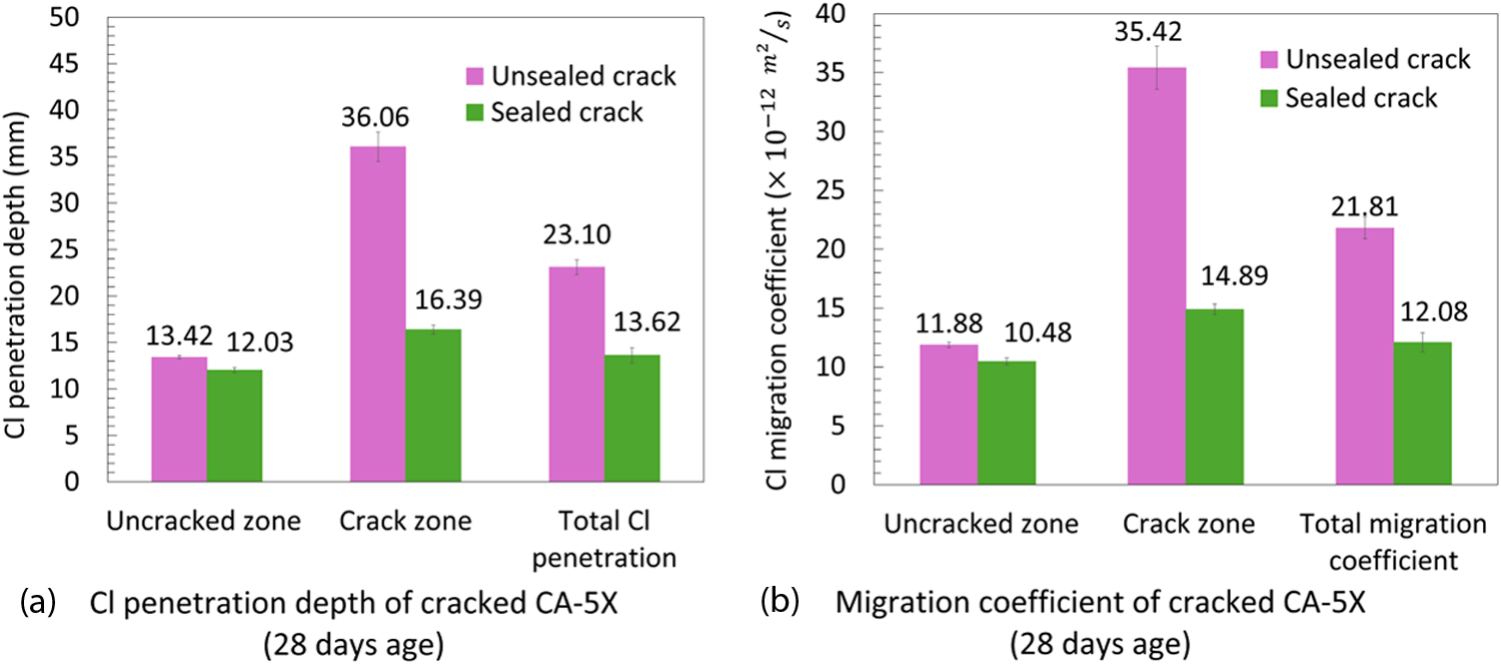
Average chloride penetration (a) depths and (b) migration coefficients of the crack zone, uncracked zone, and total for cracked CA-5X specimens before and after sealing at the age of 28 days. Each experiment is repeated three times.

### 3.4 Chloride Concentration Profile

After the chloride ingress penetration depths were measured, the concrete specimens were ground to determine their chloride concentration profile. In cracked concrete, the ground area included crack and uncracked zones. The chloride concentrations of uncracked, unsealed cracked, and sealed cracked concrete from the RCPT are shown in Figure 12. The chloride concentration reduces sharply in depth with the addition of the CA enzyme to the concrete mix (Figure 12c,12d) compared to that of the control specimens without the CA enzyme (Figure 12b). The average chloride concentration of control specimens is around 15 and 4 times larger than the CA-5X and CA-10X specimens at the deepest powdered layer (18–20 mm). This increase in chloride concentration clearly shows the beneficial impact of using CA in concrete in reducing chloride penetration.

**Figure 12:**
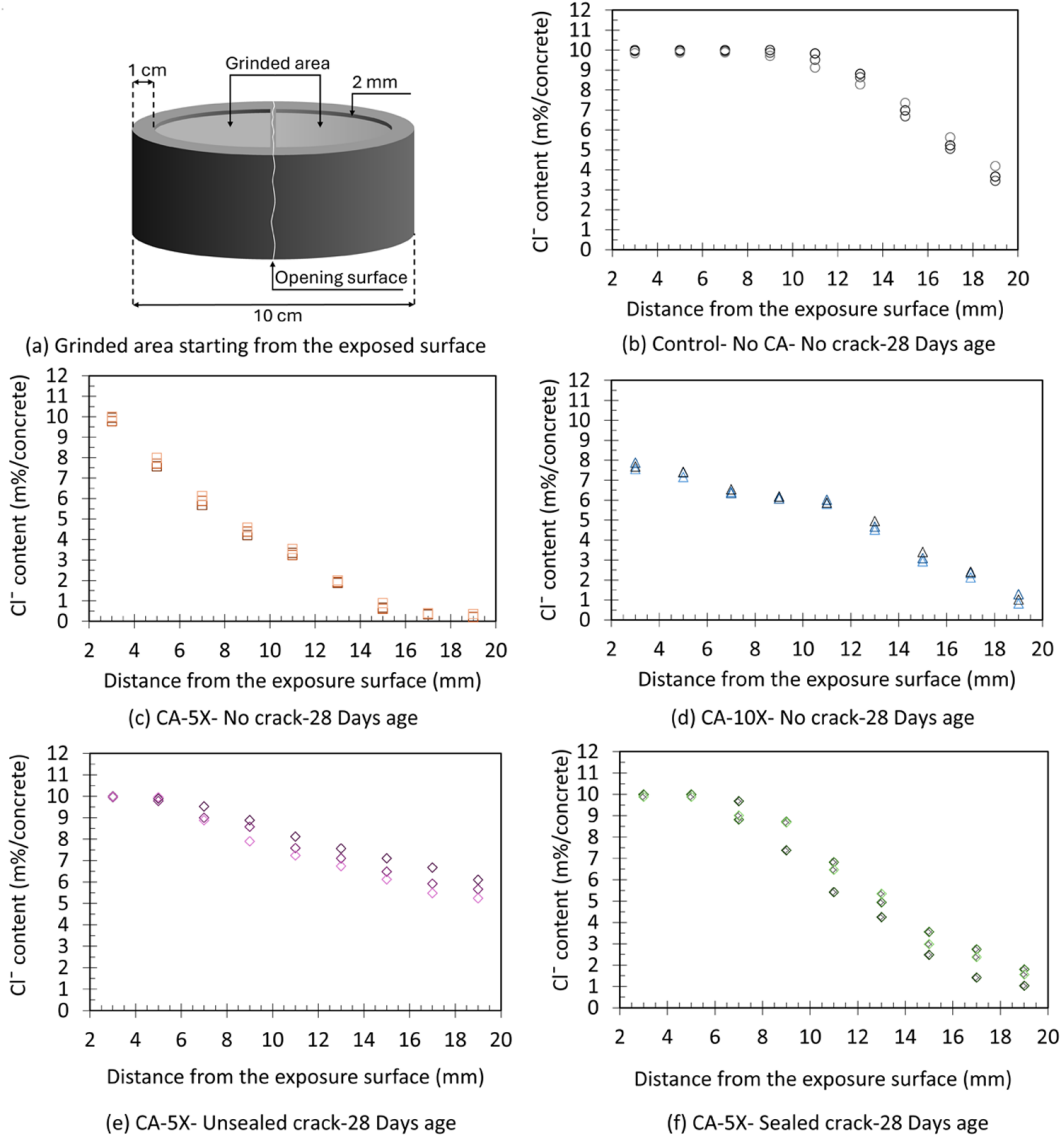
(a) Schematic of a representative 2 mm layer grinding from the exposed surface. Profiles of chloride content of RCPT specimens at 2–4, 4–6, 6–8, 8–10, 10–12, 12–14, 14–16, 16–18, and 18–20 mm depths. (b) Uncracked control concrete specimens, (c) uncracked CA-5X specimens, (d) uncracked CA-10X specimens, (e) unsealed cracked CA-5X specimens, and (f) sealed cracked CA-5X at 28 days of age.

Crack provides a more accessible path for transporting chloride ions in concrete, leading to a high concentration at the concrete depth (Figure 12e). At an 18–20 mm deep layer, the average chloride concentration of unsealed cracked CA-5X specimens is 23 times higher than the uncracked CA-5x specimens. At the same depth, the chloride concentration of sealed cracked CA-5X is more scattered than the others since crack sealing in depth can be heterogeneous. It should be noted that sealing the crack drops the chloride concentration in depth compared with unsealed cracked specimens, and its value was similar to that of the uncracked CA-10X specimens (Figure 12f).

### 3.5 Concrete Characterization

The SEM/EDX micrographs for an uncracked CA-5X concrete specimen after RCPTs are presented in Figure 13. The main elements present in Friedel’s salt are reported in the EDX data. The analysis focused on two specific locations: (a) the area that chloride ions did not reach (at 30–40 mm depth) and (0.1% Cl), and (b) the area that was reached by the chloride at 2–10 mm depth (4.6% Cl). The existence of C–S–H gel, Ca­(OH)_2_ (CH), and ettringite produced during the cement hydration is observed and indicated with yellow arrows in Figure 13a. In a comparison of the two areas (Figure 13a,13b), more ettringite and calcium hydroxide were found in the areas without chloride ions. We believe chloride ions react with the C_3_A phase according to eqs 5 and 6, forming Friedel’s salts in the areas accessed by chloride. Apart from Friedel’s salt, some chloride ions are bound by the C–S–H gels (Figure 13b,13d).^40^ This reaction typically occurs in the regions where chloride ions penetrate the concrete, leading to the formation of Friedel’s salt, altering the original microstructure, and potentially impacting the durability properties over time.

5 $$\mathrm{Ca}(\mathrm{OH}{)}_{2}+2\mathrm{NaCl}\to {\mathrm{CaCl}}_{2}+2{\mathrm{Na}}^{+}+2{\mathrm{OH}}^{-}$$

6 $$C_{3}A+{\mathrm{CaCl}}_{2}+10H_{2}O\to C_{3}A\cdot {\mathrm{CaCl}}_{2}\cdot 10H_{2}O$$

**Figure 13:**
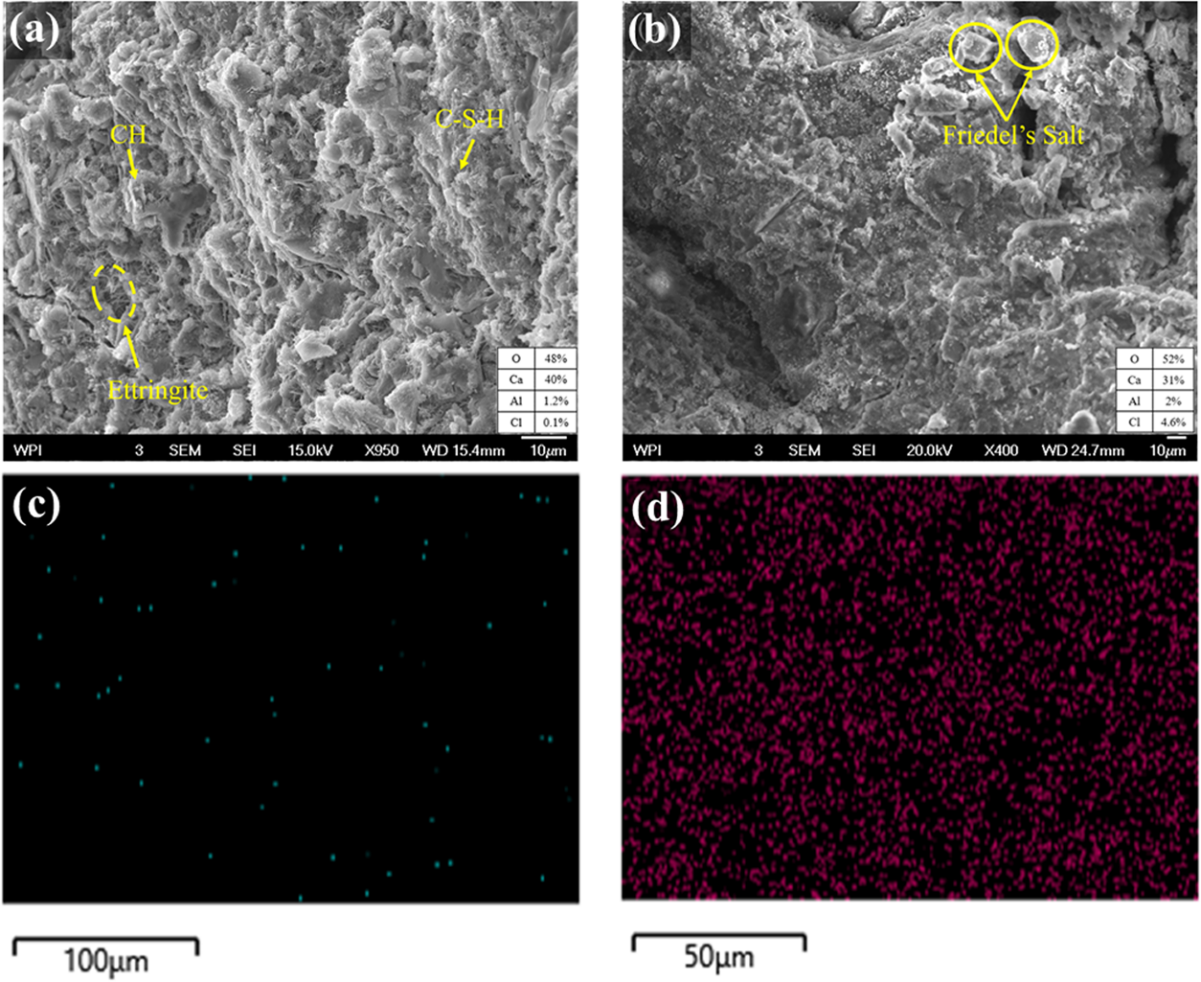
Scanning electron microscopy (SEM) images of (a) an area that chloride did not reach during the test and (b) an area that chloride penetrated during the test. (c, d) The corresponding energy-dispersive X-ray (EDX) analysis for uncracked control concrete without CA at 28 days of age. The formation of Friedel’s salt can be observed in the penetrated areas.

XRD analysis was performed to detect the Friedel’s salt in the area reached by the chloride at 2–4 mm depth and compared with the XRD analysis at 19–21 mm depth, which was not accessed by the Friedel’s salt, apart from the exposed surface to the chloride attack in RCPT. The XRD analysis further confirmed Friedel’s salt formation in the chloride-exposed zone (Figure 14), which is aligned with the SEM-EDX results at the exact locations specified on the specimens in Figure 13. Because the RCPT test involves a short-term evaluation process, it does not provide sufficient time to observe the full impact of carbonic anhydrase (CA) on the formation of Friedel’s salt. Since the formation of Friedel’s salt can take longer to develop, a long-term study is needed to assess how CA influences this process more clearly. Our future research will focus on this aspect, allowing for a more thorough investigation of the long-term effects of CA on Friedel’s salt formation and overall concrete durability.

**Figure 14:**
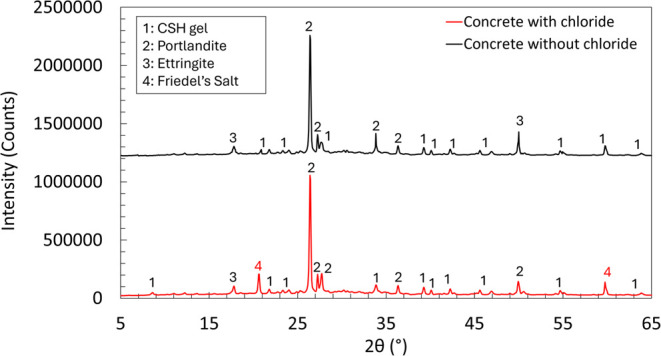
X-ray diffraction (XRD) of concrete powder in a 2–4 mm depth from the exposed surface, penetrated by the chloride after the RCPT (red), and concrete powder in a 19–21 mm depth from the exposed surface where chloride did not reach during the RCPT (black).

Studying the microstructure of concrete exposed to chloride attack is especially critical in the context of climate change, where rising sea levels and increased use of deicing salts can intensify chloride exposure in various regions. The incorporation of carbonic anhydrase offers a promising approach to enhancing concrete’s resilience against such environmental challenges. The microstructural analysis findings align with the observed deterioration patterns, revealing that higher concentrations of chloride ions correlate with an increased likelihood of corrosion-related damage. This is particularly concerning in environments exposed to elevated chloride levels, such as coastal areas or regions where deicing salts are commonly used. While Friedel’s salt can sequester some chlorides, it also marks a critical threshold beyond which the structural integrity of concrete can be significantly compromised. Ultimately, by leveraging the benefits of carbonic anhydrase, we can optimize the performance of concrete in chloride-laden environments, ensuring that structures maintain their integrity and functionality over time. This knowledge contributes to the development of sustainable infrastructure that is better equipped to adapt to evolving climatic conditions and provides long-lasting value.

### 3.6 Service Life Prediction

Approximately 30 billion tonnes of concrete are used each year worldwide, and 600 kg of CO_2_ is emitted for every tonne of cement produced.^4^ The Sustainability of a reinforced concrete structure is a direct function of its service life, and up to around 80% of corrosion-related deterioration in concrete structures can be attributed to chloride attack.^41^ A concrete structure’s “service life” is defined as the expected period during which the structure or part of it can be used for its intended purpose with regular maintenance but without significant repairs. As infrastructure owners increasingly demand longer service lives and sustainable infrastructures, ensuring these criteria are met throughout the structure’s lifespan will be crucial for future reinforced concrete (RC) design and maintenance. Service life prediction refers to the ability to quantitatively estimate, with a specified level of reliability, how long a structure will effectively serve its intended function without reaching serviceability or durability limit states. The required service life should be established by the owner, taking into account the interests of various stakeholders, including users, concessionaires, and society as a whole. When defining the design service life, it is essential to establish target reliability levels and determine what signifies the end of service life for the structure or its components.^42^ Ultimately, the goal of service life prediction is to ensure the provision and management of infrastructure that maximizes its economic and functional value.

The chloride migration coefficient is used for predicting the service life of concrete by means of the model proposed in the fédération internationale du béton (FIB) model code for service life design.^43^ This model is based on Fick’s second law, and the service life of enzymatic self-sealing concrete is predicted based on the limit-state eq 7

7 $$t={\left[{\left(\frac{2}{c-\Delta x}erf^{-1}\left(1-\left(\frac{C_{crit}-C_{0}}{C_{S,\Delta x}-C_{0}}\right)\right)\right)}^{-2}\cdot \frac{1}{k_{e}\cdot D_{\mathrm{RCM},0}k_{t}\cdot t_{0}^{a}}\right]}^{1/1-a}$$

where *t* is time (years), *c* is the concrete cover (mm), Δ*x* is the depth of convection zone, *C*_crit_ is the design value of the critical chloride concentration (wt.-%c), *C*_0_ is the design value of initial chloride content of concrete (wt.-%c), *C*_S,Δ*x*_ is the chloride content at a depth Δ*x* (wt.-%c) and a certain point of time *t*, *k*_e_ is the environmental transfer variable (−), *D*_RCM,0_ is the rapid chloride migration coefficient calculating based on the RCPT results (mm^2^/a), *k*_*t*_ is the transfer parameter (−), and *t*_0_ is the reference point of time and *a* is the aging exponent (−). Table 4 shows the parameters used to predict the service life of concrete.

**Table 4:** Parameters for Service Life Prediction

*C*	Δ*x*	*C*_crit_	*C*_0_	*C*_S,Δ*x*_	*t*_0_	*k*_e_	*k*_*t*_	*a*
(mm)	(mm)	(wt.-%c)	(wt.-%c)	(wt.-%c)	(year)			
50	0	0.6	0.06	3	0.0767	1.26	1	0.3

To predict the service life of Portland cement concrete without using additives, normally 28 days age of the concrete is used as a reference time *t*_0_ since over 90% of final compressive strength is reached in Portland cement concrete with a typical water/cement (W/C) ratio.^44^

For each group of tested specimens, the average value of the chloride migration coefficient is calculated based on the NTBUILD-492 ^12^ as *D*_RCM,0_. The mean value of aging exponent *a* of 0.3 is chosen based on the W/C and type of cement, and the transfer parameter *k*_*t*_ is set to 1. The reason that the environmental transfer variable, *k*_e_, has been introduced is to consider the influence of the temperature of the structural element or ambient air (*T*_real_). Eq 8 is used to calculate *k*_e_.

8 $$k_{e}=\exp\left[b_{e}\left(\frac{1}{T_{\mathrm{ref}}}-\frac{1}{T_{\mathrm{real}}}\right)\right]$$

The mean value of regression variable *b*_e_ is 4800, and the standard test temperature *T*_real_ is 293 K based on the FIB model. The ambient air temperature *T*_ref_ is 317 K. The initial chloride content *C*_0_ is 0.06%. It is measured through grinding before running the RCPT and assumed to be uniform over the whole concrete cross-section. It is assumed that the structure is submerged, so *C*_S,Δ*x*_ equals the chloride saturation concentration^43,45^ and Δ*x* is zero. The mean value of 0.6% is considered for *C*_crit_. The concrete cover is assumed to be the same as the thickness of tested specimens in the RCPT (Table 4).

Based on FIB 34,^43^ the predicted service life of the control specimen is 262 years. This baseline establishes a reference point for evaluating the impact of CA-5X and CA-10X. Using CA-5X and CA-10X times in concrete extends the service life to 634 and 521 years, respectively, in uncracked concrete. This enhancement is noteworthy as it demonstrates the potential for modern materials to improve the longevity of concrete structures substantially. However, the analysis reveals a critical challenge: the presence of cracks drastically reduces the service life. Cracks facilitate the ingress of chloride ions, which can quickly reach harmful concentrations at the surface of the rebar, compromising structural integrity. This highlights the importance of not only using advanced materials but also ensuring that the concrete minimizes cracking throughout its service life. The ability to seal these cracks presents an innovative solution. When cracks are sealed, the service life in those areas can be restored to 327 years. This recovery underscores the potential for integrating self-sealing technologies into concrete design, enhancing durability and sustainability.

The results show that a low dosage of the enzyme (CA-5X) can extend the service life of uncracked concrete by approximately 2.4 times compared to that of control concrete and recover the service life at the sealed crack zone in cracked concrete, which is a significant improvement. If a structure lasts 2.4 times longer before requiring major repairs or replacement, the frequency of cement production and the associated CO_2_ emissions over the structure’s lifetime are significantly reduced. For example, if we assume that building material and construction contribute 12% of CO_2_ emissions from the concrete industry, extending the 2.4 times service life of concrete structures using carbonic anhydrase results in a reduction of 0.4% of the CO_2_ emissions. Therefore, enhancing the long-term durability and repairing the damaged concrete can significantly impact its overall sustainability. This holistic approach not only addresses current performance but also anticipates future challenges, ultimately contributing to more sustainable and resilient infrastructure systems.

Corrosion-induced cracking typically originates internally as corrosion products expand and induce tensile stresses that propagate cracks outward. Conversely, external cracks form at the surface owing to tensile stresses and extend inward toward the reinforced concrete. Sealing these surface cracks is critical for minimizing chloride ingress and delaying the onset of corrosion at the rebar surface. Environmental conditions, particularly RH and hydration, play a vital role in the long-term effectiveness of this method. These factors must be carefully considered when the technology is applied under field conditions, and future studies should explore the effects of varying hydration states to optimize the performance.

## 4 Conclusions

We experimentally studied the resistance of enzymatic self-sealing concrete to chloride ingress by the rapid chloride penetration tests on uncracked concrete specimens at 14 and 28 days of age and cracked concrete specimens before and after sealing at 28 days. The microstructural analysis of the specimens revealed Friedel’s salt in the area reached by the chloride ions, and the concentration of Friedel’s salt was compared for regions with and without chloride ions. Finally, service life was predicted based on the international standard at both uncracked and cracked states at 28 days of age. The following conclusions can be drawn based on the obtained results from this study:

The addition of carbonic anhydrase (CA) significantly influences the diffusion of chloride ions at an early age (14 days) of concrete and improves its resistance to chloride ingress. In addition, using a higher dosage of CA (CA-10X) does not negatively affect the chloride penetration of concrete compared with the control specimens without the CA enzyme.

An increase in the curing period from 14 to 28 days considerably reduced the resistance of concrete to chloride ingress. Additionally, chloride ion diffusion was significantly decreased by incorporating CA in concrete. The chloride migration coefficient was reduced by 46% by using a lower dosage of CA (CA-5X) compared to the control 28-day specimens.

The presence of a crack leads to a significant increase of chloride ion migration in concrete and diminishes its resistance to chloride ingress at the crack zone. Sealing the crack, with 100% sealing efficiency of the carbonic anhydrase at the surface of the concrete, can restore concrete’s durability to its uncracked level and considerably reduce the chloride penetration at the crack zone.

Incorporating carbonic anhydrase in different dosages, CA-5X and CA-10X, can significantly enhance the service life, extending it beyond traditional expectations. Moreover, sealing the crack in concrete containing a lower dosage of CA, CA-5X, enables the recovery of service life in the crack zones of cracked concrete. These findings represent significant advancements in concrete sustainability as the second largest industrial emitter. Moreover, full implementation of this innovation can lead to a reduction of 0.4% in entire CO_2_ emissions, contributing to more sustainable infrastructure.
